# ARHGAP21 Acts as an Inhibitor of the Glucose-Stimulated Insulin Secretion Process

**DOI:** 10.3389/fendo.2020.599165

**Published:** 2020-11-26

**Authors:** Sandra M. Ferreira, José M. Costa-Júnior, Mirian A. Kurauti, Nayara C. Leite, Fernanda Ortis, Luiz F. Rezende, Helena C. Barbosa, Antonio C. Boschero, Gustavo J. Santos

**Affiliations:** ^1^Obestity and Comorbidities Research Center/Biology Institute, University State of Campinas (UNICAMP), Campinas, Brazil; ^2^Departament Physiological Sciences, University State of Maringá (UEM), Maringá, Brazil; ^3^Department of Cellular Biology and Development, Institute of Biomedical Sciences, University State of São Paulo (USP), São Paulo, Brazil; ^4^Departament of Physiopathology, University State of Montes Claros (UNIMONTES), Montes Claros, Brazil; ^5^Departament of Physiological Sciences, Center for Biological Sciences, University Federal of Santa Catarina (UFSC), Florianópolis, Brazil

**Keywords:** ARHGAP21, insulin secretion, calcium influx, type 2 diabetes, Ob/Ob mice

## Abstract

ARHGAP21 is a RhoGAP protein implicated in the modulation of insulin secretion and energy metabolism. ARHGAP21 transient-inhibition increase glucose-stimulated insulin secretion (GSIS) in neonatal islets; however, ARHGAP21 heterozygote mice have a reduced insulin secretion. These discrepancies are not totally understood, and it might be related to functional maturation of beta cells and peripheral sensitivity. Here, we investigated the real ARHGAP21 role in the insulin secretion process using an adult mouse model of acute ARHGAP21 inhibition, induced by antisense. After ARHGAP21 knockdown induction by antisense injection in 60-day old male mice, we investigated glucose and insulin tolerance test, glucose-induced insulin secretion, glucose-induced intracellular calcium dynamics, and gene expression. Our results showed that ARHGAP21 acts negatively in the GSIS of adult islet. This effect seems to be due to the modulation of important points of insulin secretion process, such as the energy metabolism (PGC1α), Ca^2+^ signalization (SYTVII), granule-extrusion (SNAP25), and cell-cell interaction (CX36). Therefore, based on these finds, ARHGAP21 may be an important target in Diabetes Mellitus (DM) treatment.

**Graphical Abstract:**
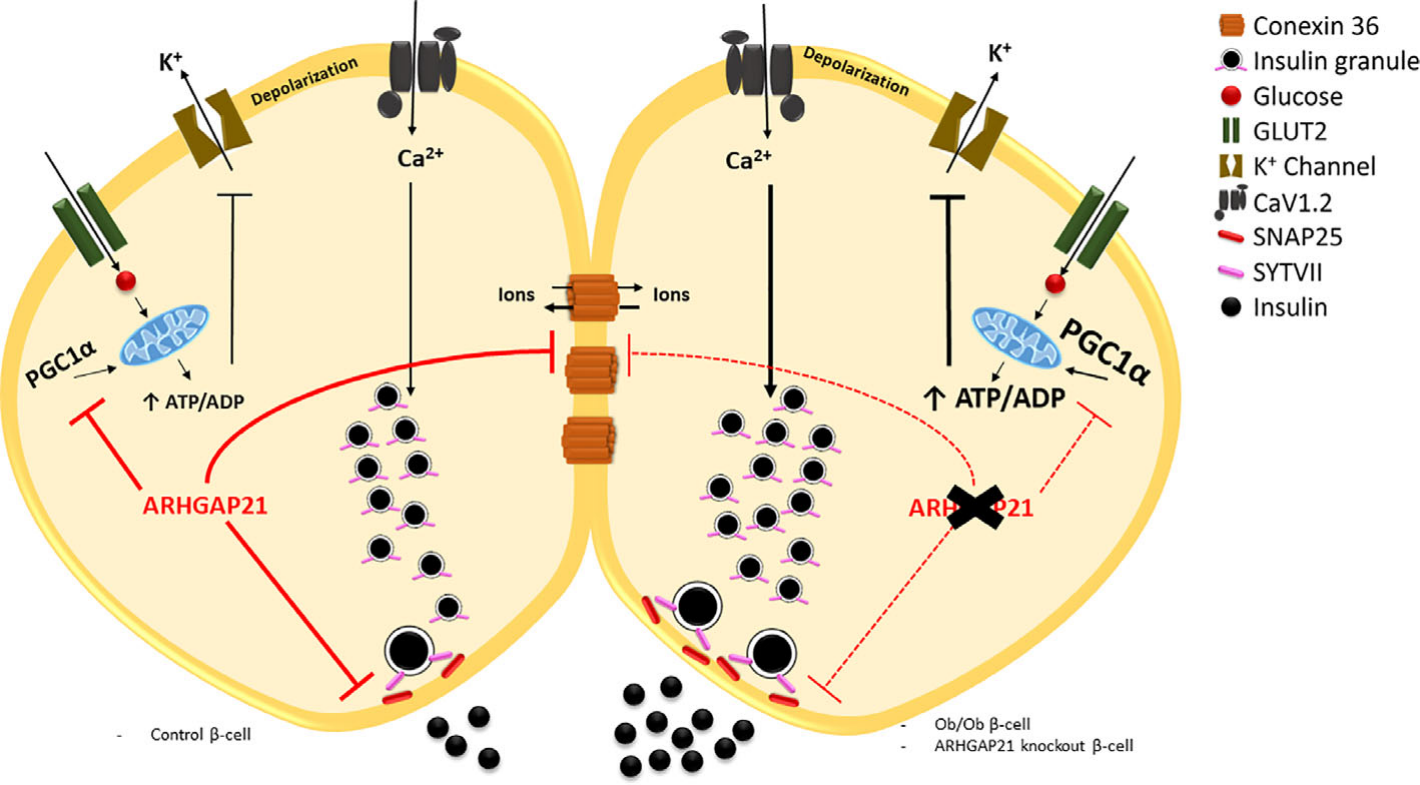
ARHGAP21 reduces glucose-induced insulin secretion (GSIS) through the modulation of the expression of genes that encoding crucial proteins to the GSIS process, such as PGC1α, CX36, SYTVII, and SNAP25. In a scenario with a reduction of ARHGAP21 expression (ex. Ob/Ob and AS-induced knockdown) it is observed an increase in the expression of these genes and, consequently in the GSIS.

## Introduction

Diabetes Mellitus (DM) is a highly prevalent disease, and pancreatic islet dysfunction is crucial for its development (1–3). Thus, investigating proteins and process that can mitigate beta cell overload and dysfunction may reveal new targets for better management of this pathology. In this context, our group has explored the role of ARGHAP21 (a RhoGAP protein), that has been implicated in cancer due to its suppressor tumor function, (4, 5), and in the glycemic and insulin homeostasis (6–9).

We previously demonstrated that ARHGAP21 inhibition increases insulin secretion in neonatal isolated pancreatic islet (6). However, we also reported that isolated pancreatic islet from transgenic heterozygote mice (with around 50% less expression of ARHGAP21) present a reduction in the insulin secretion when compared to the wild-type mice fed on the same diet (7). The discrepancies between both studies could be explained, at least in part, by the fact that neonate islet are functionally immature, presenting significant physiology differences compared to the islets from adult mice. In addition, the reduced insulin secretion found in our study using transgenic adult mice, seems to be due to a compensatory response to the improvement of insulin action, observed in the peripheral tissues from ARHGAP21 heterozygote mice (7). In the present study, we aim to investigate the real role of ARHGAP21 in the insulin secretion process using an acute ARHGAP21 inhibition adult mice model excluding both neonatal and the insulin sensitivity effect. In addition, we measured the ARHGAP21 expression in the pancreatic islet of animal models known to present hyper (B6.V-Lep^ob^/JUnib) and hypo (endurance-trained mice) insulin secretion.

Here, we observed that B6.V-Lep^ob^/JUnib mice (Ob/Ob), an animal model known to secrets a huge amount of insulin, display reduced ARHGAP21 expression in their pancreatic islets in comparison to a lean mice. In contrast, an animal model known to secrets a low amount of insulin (endurance-trained mice) showed the opposite results, presenting high ARHGAP21 content compared to control group. These findings suggest that our target protein acts as a negative regulator of insulin secretion. This observed phenomenon was corroborated by the fact that adult islet from ARHGAP21 knockdown mice showed increased glucose-stimulated insulin secretion (GSIS). This is probably due to upregulation of proteins involved in the lipid metabolism (PGC1α), Ca^2+^ sensibility (SYTVII), granule extrusion (SNAP25) and cell-cell interactions (CX36).

Taken together, these findings confirm the involvement of ARHGAP21 over insulin/glucose homeostasis. The discovered of this important role of ARHGAP21 as a negative regulator of insulin secretion may provide a target for therapies to DM management. However, further studies are necessary to better understand by which mechanism ARHGAP21 acts in metabolic diseases, such as obesity and diabetes.

## Methods

### Reagents

The oligonucleotides (antisense-ARHGAP21 and Mismatch) and primers were purchased from Sigma Adrich (St. Louis, MO, USA). Fura-2 acetoxymethyl ester was acquired from Life Technologies (Carlsbad, CA, USA). Anti-ARHGAP21 and anti-GAPDH were acquired from Santa Cruz Biotechnology (Santa Cruz, CA, USA). The secondary antibody goat-anti-rabbit was obtained from Thermo Scientific (Waltham, MA, USA).

### Animals

All experiments were approved by the Committee for Ethics in Animal Experimentation of the State University of Campinas (CEUA/IB/UNICAMP – 2507-1).

- For experiments involving acute inhibition of the ARHGAP21: C57BL/6, aged 60 days from the State University of Campinas animal facilities were used in the experiments. Mice were maintained in appropriate cages (4 animals/cages) on a 12 h light-dark cycle at 20–21°C with controlled humidity, with access to food and water ad libitum. They received daily intraperitoneally (i.p.) injections of 0.15 nmol/g of anti-ARHGAP21 oligonucleotide (AS) or Mismatch (CTL) in Tris-EDTA (Veichel) buffer for 3 consecutive days, and all experiments were performed 24 h after the last injection. Oligonucleotides utilized in this study: Anti-ARHGAP21: mC*mU*mU*mU*C*C*T*C*C*T*C*T*G*T*mU*mU*mC*mC and Mismatch: mC*mU*mU*mU*C*T*A*C*C*T*C*A*G*T*mU*mU*mC*mC.
- For experiments involving hypo insulin-secreting mice: 8–12 weeks old male Swiss mice, acquired from the State University of Campinas, were maintained on a 12 h light-dark cycle at 20–21°C, with controlled humidity during the entire experiment and fed with a standard CHOW diet and offered tap water *ad libitum*. Mice were randomly assigned to a sedentary control group (CTL), which limited to typical movement inside the cages, and the endurance-training group (T). As previously described (10), the endurance-training group performed a four-week training protocol for running on the treadmill. During the four weeks, mice ran five days per week for 1 h each day. During the first two weeks of training, the intensity was set at 70% VO_2_ max, and in the last two weeks, the intensity was set at 80% VO_2_ max. Twenty-four hours after the last training session, the mice were killed and the islets were isolated.
- For experiments involving hyper insulin-secreting mice: 60 days old male B6.V-Lep^ob^/JUnib (Ob/Ob) and lean males C57BL/6J of matched age were acquired from the State University of Campinas, the mice were killed and the islets were isolated.

### Islet Isolation and Insulin Secretion

Mice were killed by CO_2_ gas exposure and the pancreas was inflated with a Hanks buffer (137 mM NaCl, 5.5 mM KCl, 4.5 mM NaHCO_3_, 0.4 mM KH_2_PO_4_, 0.4 mM Na_2_HPO_4_, 0.8 mM MgSO_4_, 1.5 mM CaCl_2_, pH 7.4) containing 0.8 mg/ml of collagenase. After removal, the inflated pancreas was incubated for 17 min at 37°C. After the incubation, the solution was shaken gently to complete dissociation of the islets and washed with cold Hanks to stop the enzyme activity. To confirm the inhibition of ARHGAP21, pancreatic islets were lysed in an urea anti-protease/anti-phosphatase buffer (7 mM urea, 2 M thiourea, 5 mM EDTA, 1 mM sodium fluoride, 1 mM orthovanadate, 1 mM pyrophosphate and 2 mM phenylmethylsulfonyl fluoride and 1% Triton-X100), and ARHGAP21 expression was evaluated by western blotting. For GSIS analysis, the islets were collected one-by-one and pre-incubated in Krebs bicarbonate buffer (KRBB - 115 mmol/l NaCl, 5 mmol/l KCl, 2.56 mmol/l CaCl_2_, 1 mmol/l MgCl_2_, 10 mmol/l NaHCO_3_, 15 mmol/l HEPES, pH 7.4) supplemented with 5.6 mM glucose and 0.2 g/L BSA for 1 h at 37°C, and were, subsequently, incubated in a similar buffer with 2.8, 5.6, 8.3, 11.1, 16.8, or 22.2 mM of glucose or 30 mM of KCl for 1 h at 37°C. After incubation, the supernatant fraction was collected, and the secreted insulin was measured by Radioimmunoassay (RIA). For total insulin content, after the incubation period, the islets were collected and transferred to 1.5 ml tubes. Deionized water (1 ml) was then added to the samples, followed by disruption of the pancreatic cells using a Polytron PT 1200 C homogenizer (Brinkmann Instruments, NY, USA). The total insulin content was also measured by RIA. The insulin secretion was normalized by the total insulin content.

### Plasma Insulin Levels

10 hours fasting mice received an i.p. glucose solution at a concentration of 1 g/kg in 0.9% w/v of NaCl solution. The blood glucose was measured at baseline (before glucose administration; 0 min) and after 15 and 60 min of the injection. Glucose was evaluated with glucose strips on an Accu-Chek Performa II instrument (Roche). Blood samples (100 µl) were collected from the tail immediately before the injection (0 min), and at 15 and 60 min following the injection to determine the concentrations of insulin that were measured by RIA.

### Hyperinsulinemic-Euglycemic Clamp

Fasted mice (8 h) were anesthetized (ketamine, xylazine and 0.9% w/v NaCl solution – 1.5:2:1.5) and received a catheter in the right carotid. After surgery, mice received an i.p. insulin-bolus injection (400 mU/Kg). During the test, it was infused continuously 30 mU/Kg/min of insulin and 5% glucose solution. This infusion was regulated until the mouse maintains normoglycemia for 30 min. Glucose was evaluated every 5 min with glucose strips on an Accu-Chek Performa II instrument (Roche). The glucose infusion rate was determinate by calculation 1000/bodyweight x infused glucose.

### Intracellular Calcium Concentration

Islets were incubated in KRB buffer containing 5.6 mM glucose at 37°C for 2 h. During the last hour of incubation, islets were loaded with 5 µM of the Ca^2+^-sensitive dye Fura-2 acetoxymethylesther (AM). Afterward, single islets were placed inside a thermostatically-regulated chamber (37°C) over poly-L-lysine-treated glass coverslips and perfused with a BSA-free KRB buffer containing 2.8 or 22.2 mM glucose or 30 mM KCl. Fura-2AM loaded islets were imaged using an inverted epifluorescence microscope (Nikon Eclipse TE200, Tokyo, Japan). A ratio image was acquired every 3 s with a Cool One camera (Photon Technology International, NJ, USA) using a dual filter wheel equipped with 340, 380, and 10 nm bandpass filters, and a range of neutral density filters (Photon Technology International, NJ, USA). Data were acquired using the Image Master Version 5.0 software (Photon Technology International, NJ, USA).

### Quantitative Real-Time PCR

Pancreatic islets mRNA was extracted by RNeasy kit (Qiagen, Cat. #74007), then 200 ng of purified mRNA was used to synthesize the cDNA (High-Capacity cDNA reverse transcription kit, Applied Biosystems, Foster City, CA). The primers were designed and tested against the *Mus musculus* genome (GenBank). Relative quantification was performed using the Step-one real-time PCR system (Applied Biosystems). The relative quantities of the target transcripts were calculated from duplicate samples (2^ΔΔCT^), the data were normalized against the endogenous control GAPDH. The studied genes were as follows: NKX6.1, PDX1, MAFA, HFN4α, INS1, INS2, CX 36, GLUT2, GCK, PGC1α, SYT VII, VAMP2, SYNTAXIN 1A, SNAP25, CYCLIN D2, and CDK4. The primer sequences are available in the Supplementary.

### Western Blotting

Protein concentration was determined by the Bradford method using bovine serum albumin (BSA) as standard. Fifty (50) μg of the lysate was boiled in SDS loading buffer, applied on 10% SDS-PAGE and transferred to nitrocellulose membranes, subsequently blocked in TBS buffer (10 mmol/l Tris base, 150 mmol/l NaCl and 0.25% of Tween 20) containing 5% BSA powder for 1 h at room temperature. Membranes were then incubated with primary antibodies at 4°C, overnight, and secondary antibodies for 1 h at room temperature. The intensities of the protein bands were detected using a LAS-3000 CCD camera, and quantification was performed using densitometry (ImageJ, Bethesda, USA). The densitometry values of target protein were normalized by GAPDH bands intensities. The antibodies used in this article can be found in Supplementary Material.

### Statistical Analysis

The data are expressed as the mean ± SEM. Statistical analyses were performed using Student’s t-test and ANOVA two-away when necessary. P ≤0.05 was considered statistically significant.

## Results

### Expression of ARHGAP21 in Mice Models With Altered Insulin Secretion

**Figure 1:**
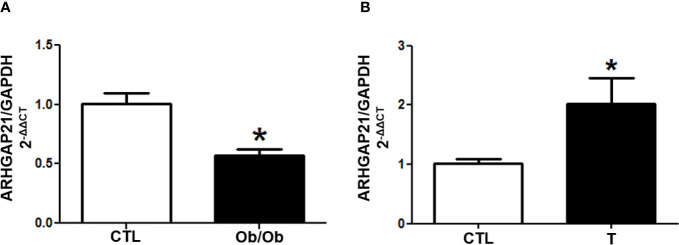
ARHGAP21 expression in islet from hyper- and hypo-secreting insulin animal model. ARHGAP21 expression was evaluated by RT-qPCR in islet from **(A)** B6.V-Lep^ob^/JUnib mice (Ob/Ob) and **(B)** endurance-trained mice (T), compared to the CTL group. In islets from Ob/Ob mice ARHGAP21 was reduced by 40%, and 2 times increased in islets from trained mice (n = 3–4). Data are presented as the mean ± SEM. *P ≤ 0.05 vs CTL. CTL, control mice; Ob/Ob, B6.V-Lep^ob^/JUnib mice; T, endurance training; SEM, standard error of the mean.

We investigated the gene expression of ARHGAP21 in islets from Ob/Ob mice (B6.V-Lep^ob^/JUnib), that secrete high amount of insulin (11), and from endurance-trained mice who display a reduced GSIS (12). Interestingly, we found that in the pancreatic islet from Ob/Ob mice the ARHGAP21 expression was significantly reduced (**Figure 1A**), whereas in the pancreatic islet from endurance-trained mice it was increased, compared to the control (CTL) (**Figure 1B**). These results corroborate the idea that ARHGAP21 may also modulate insulin secretion in adult mice.

### Islet ARHGAP21 Expression, Plasma Insulin Level, and Hyperinsulinemic-Euglycemic Clamp in ARHGAP21 Knockdown Mice

**Figure 2:**
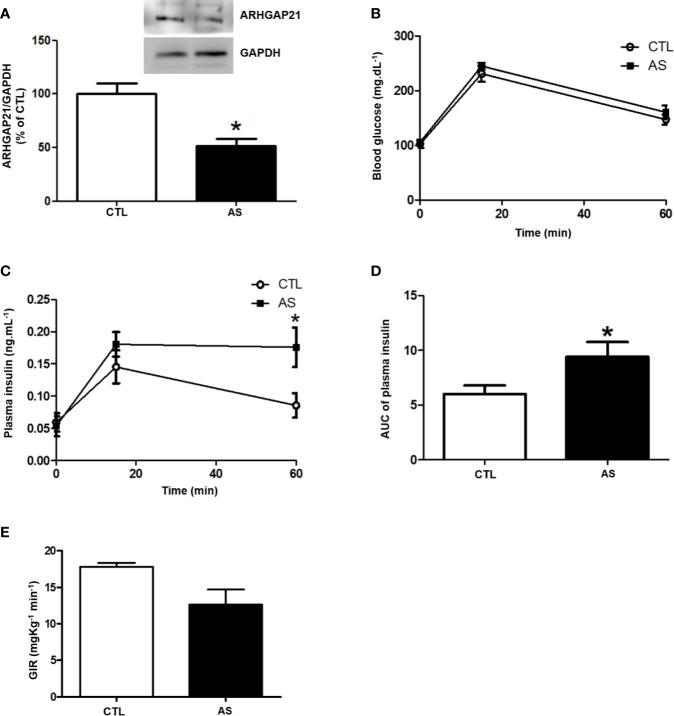
ARHGAP21 knockdown effects on pancreatic islets ARHGAP21 expression, glucose tolerance, plasma insulin during ipGTT, and insulin sensitivity. Adult mice received intraperitoneal administrations of antisense anti-ARHGAP21 for three consecutive days, and the experiments were performed 24 h after the last administration. **(A)** Pancreatic islets ARHGAP21 protein expression by western blotting. **(B)** Blood glucose before (0 min), 15, and 60 min after an intraperitoneal administration of 1 g/Kg of glucose (ipGTT). **(C)** Insulin plasma before (0 min), 15, and 60 min after an intraperitoneal administration of 1 g/Kg of glucose. **(D)** AUC of the plasma insulin during ipGTT. **(E)** Hyperinsulinemic-euglycemic clamp. N = 8–16. Data are presented as the mean ± SEM. *P ≤ 0.05 vs CTL. CTL, control mice; AS, mice that received intraperitoneal administrations of antisense anti-ARHGAP21; ipGTT intraperitoneal glucose tolerance test; AUC, area under the curve; SEM, standard error of the mean.

To study the role of ARHGAP21 in β-cell physiology we knockdown this protein by an antisense administration. First, to confirm the ARHGAP21 knockdown, we analyzed the ARHGAP21 protein level in C57BL/6 mice, after the administration of antisense anti-ARHGAP21 for 3 days, and we observed a reduction (by 50%) of islet ARHGAP21 expression, compared to CTL mice islet (**Figure 2A**). Next, to investigate the role of ARHGAP21 on glucose metabolism, we evaluated glucose and insulin plasma levels before and after (15 and 60 min) glucose administration (1 g/kg) (**Figures 2B–D**). Variation of glucose plasma concentration was similar for both genotypes (**Figure 2B**), however, ARHGAP21 KO (AS) islet showed an increased insulin plasma concentration compared to islets from CTL (**Figures 2C, D**). To exclude any bias of the insulin action in peripheral tissues, we evaluated the insulin sensitivity by a hyperinsulinemic-euglycemic clamp technique and no significant alterations were observed between groups (**Figure 2E**), indicating that the effects observed in ARHGAP21 knockdown insulinemia is a result of the modulation in pancreatic islet function and not of the insulin sensitivity.

### Glucose and KCl-Stimulated Insulin Secretion in Isolated Islet

**Figure 3:**
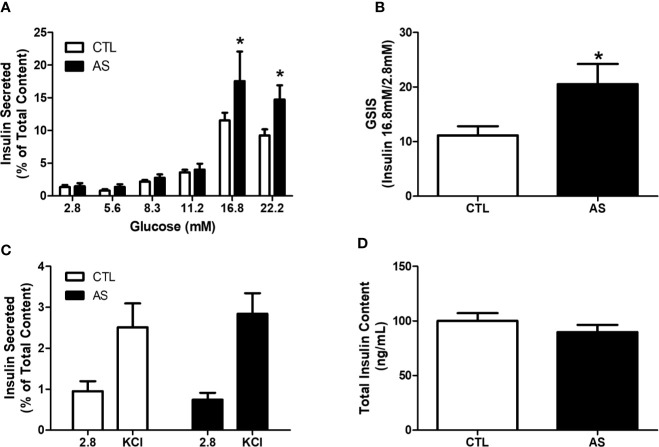
ARHGAP21 knockdown effects on glucose and KCl-stimulated insulin secretion and total insulin content. Adult mice received intraperitoneal administrations of antisense anti-ARHGAP21 for three consecutive days, and pancreatic islets were isolated 24 h after the last administration. **(A)** Insulin secreted after 1 h incubation with 2.8, 5.6, 11.2, 16.8, and 22.2 mM of glucose. **(B)** The ratio of insulin secreted between 2.8 and 16.8 mM glucose. **(C)** Insulin secreted after 1 h incubation with 2.8 mM of glucose and 30 mM of KCl. **(D)** Total insulin content of pancreatic islets. n = 7–8. Data are presented as the mean ± SEM. *P ≤ 0.05 vs CTL. CTL, control mice; AS, mice that received intraperitoneal administrations of antisense anti-ARHGAP21; GSIS, glucose-stimulated insulin secretion; SEM, standard error of the mean.

To confirm that the increased insulin levels were due to the alteration of the insulin secretion process, we stimulated pancreatic islet from CTL and AS mice with several glucose concentrations (2.8, 5.6, 8.3, 11.1, 16.8, and 22.2 mM). We observed higher insulin secretion in islets from the AS group at 16.8 and 22.2 mM glucose, compared with CTL (**Figure 3A**). Of note, the increment in insulin secretion at 16.8 mM related to 2.8 mM was significantly higher in AS compared with CTL islets (**Figure 3B**), indicating an increase in the secretory function. The insulin secretion induced by KCl (30 mM) (**Figure 3C**), as well as the total insulin content (**Figure 3D**), were also investigated, and no differences was observed, compared with the respective controls.

### [Ca^2+^]_i_, and CaV1.2 and SERCA Gene Expression

**Figure 4:**
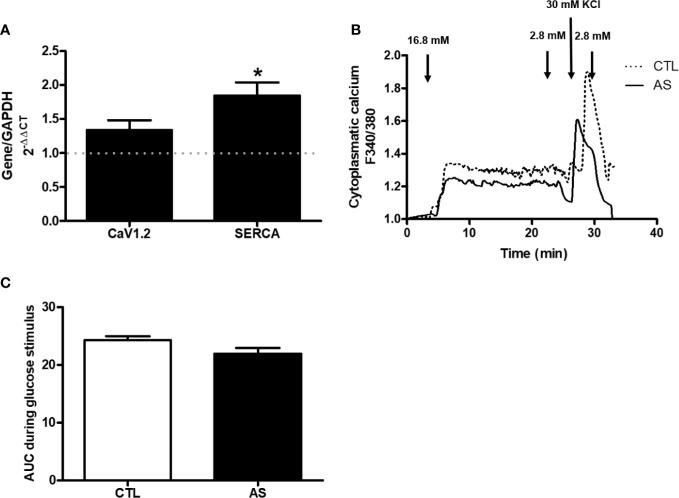
ARHGAP21 knockdown effects on calcium channels expression and on the glucose-induced calcium dynamics of pancreatic islets. Adult mice received intraperitoneal administrations of antisense anti-ARHGAP21 for three consecutive days and pancreatic islets were isolated 24 h after the last administration. **(A)** CaV1.2 and SERCA gene expression related to the CTL (dash line) **(B)** Cytoplasmic calcium after incubation with 16.8 mM glucose or 30 mM KCl. **(C)** AUC of the cytoplasmic calcium during the glucose stimulus. n = 6–8 for CaV1.2 and SERCA expression and n = 4 for cytoplasmic calcium. Data are presented as the mean ± SEM. *P ≤ 0.05 vs CTL. CTL, control mice; AS, mice that received intraperitoneal injections with antisense anti-ARHGAP21; AUC, area under the curve.

Since the level of calcium [Ca2+]i is a primary intracellular secretory signal coupling the stimulus to secretion (13–15), we addressed our focus on the gene expression of calcium channels (CaV1.2 and SERCA). We observed increased the SERCA gene expression in islets from AS mice, compared to CTL, but no significant alteration of CaV1.2 expression (**Figure 4A**). To evaluate if this alteration in SERCA expression had some effect in the glucose-induced alterations in intracellular Ca^2+^ dynamic we assessed the [Ca^2+^]_i_ in islets from both groups during a glucose stimulus. Despite the apparent difference in the glucose-induced [Ca^2+^]_I_ dynamic curve no differences where observed in the [Ca^2+^]_I_ among the genotypes, both at 16.8 mM glucose or at 30 mM KCl (**Figures 4B, C**). These results indicate that ARHGAP21 modified the expression of a gene involved with the [Ca^2+^]_i_ dynamics, but was insufficient to alter the intracellular Ca^2+^ concentration during a glucose stimulus.

### mRNA Levels of Genes Involved With Cell Maturation, Proliferation, Insulin Extrusion, and Glucose Metabolism

**Figure 5:**
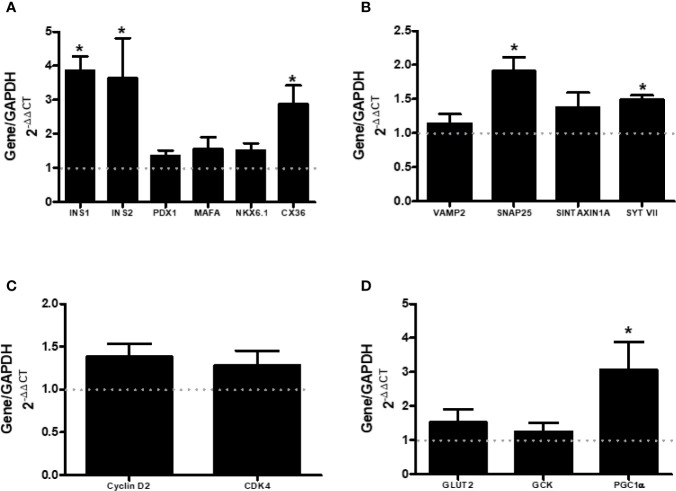
ARHGAP21 knockdown effects on mRNA level of genes that modulate maturation (INS1, INS2, PDX1, MAFA, NKX6.1, and CX36), granule extrusion (VAMP2, SNAP25, SINTAXINA1A, and SYTVII), proliferation (CYCLIN D2 and CDK4), and metabolism (GLUT2, GCK and PGC1α) in pancreatic islets. Adult mice received intraperitoneal administrations of antisense anti-ARHGAP21 for three consecutive days and pancreatic islets were isolated 24 h after the last administration. **(A)** INS1, INS2, PDX1, MAFA, NKX6.1, and CX36 gene expression related to the CTL (dash line). **(B)** VAMP2, SNAP25, SINTAXINA1A, and SYTVII gene expression related to the CTL (dash line). **(C)** CYCLIN D2 and CDK4 gene expression related to the CTL (dash line). **(D)** GLUT2, GCK and PGC1α gene expression related to the CTL (dash line). n = 4–8 Data are presented as the mean ± SEM. *P ≤ 0.05 vs CTL. CTL, control mice; AS, mice that received intraperitoneal injections with antisense anti-ARHGAP21.

We also assessed the expression of genes that encode protein involved with β-cell maturation (INS1, INS2, PDX-1, MAFA, NKX6.1, and CX36) (**Figure 5A**), β-cell proliferation (CYCLIN D2 and CDK4) (**Figure 5C**), secretory machinery (VAMP2, SNAP25, SINTAXIN 1A, and SYTVII) (**Figure 5B**), and glucose metabolism (GLUT2, GCK, and PGC1α) (**Figure 5D**). Islets from AS mice presented higher gene expression of INS1, INS2, CX36, SYTVII, and PGC1α, compared with CTL. This increased expression of genes encoding important proteins for the insulin secretion machinery may be involved in the observed increased insulin secretion (**Figure 2C**).

## Discussion

The participation of ARHGAP21 in the process of insulin secretion is poorly understood. It was demonstrated that islet from ARHGAP21-knockdown neonatal mice display higher insulin secretion in sub-stimulatory concentrations of glucose (2.8 mM) (6) and ARHGAP21 may control glucose and energy metabolism in adult mice (7, 8). Here, we show that ARHGAP21 also plays an important role in the regulation of insulin secretion in adult mice, acting as a negative controller.

To confirm this observation, we investigate the ARHGAP21 expression in islets of animal models known to display increased - Ob/Ob mice; (11) - or decreased - trained-mice; (12) - insulin secretion, in response to glucose. Interestingly, the expression of ARHGAP21 was reduced in islets from Ob/Ob mice (**Figure 1A**) and increased (**Figure 1B**) in islets from trained-mice, compared with their respective CTL. These observations indirectly support our view that ARHGAP21 is an important negative modulator of insulin secretion process.

To further explore the ARHGAP21 role in GSIS, we generated mice with 50% reduction of islet ARHGAP21 (AS) expression (**Figure 2A**). The AS mice showed hyperinsulinemia during an ipGTT (**Figures 2C, D**), and this effect was probably not due to alteration in their insulin sensitivity at peripheral tissues as confirmed by a hyperinsulinemic-euglycemic clamp experiment (**Figure 2E**). Thus, our first conclusion is that hyperinsulinemia, observed during the ipGTT in AS mice, was due to an increased insulin secretion instead of alteration in insulin sensitivity/action at insulin-target tissues. These observations were confirmed by exposing AS and CTL islets to high concentrations of glucose (16.8 and 22.2 mM glucose), where the insulin secretion was significantly higher in ARHGAP21 knockdown, compared to CTL islets (**Figure 3A**), with no differences in the total insulin content (**Figure 3D**).

Following, we centered our attention to different point of the insulin secretion steps in both AS and CTL islets. First, we evaluated if the reduction in insulin secretion in islets from ARHGAP21 knockdown was due to alterations in the state of the plasma membrane potential of the secretory cells. For this purpose, we submitted the islets to depolarizing concentrations of KCl (30 mM) and we observed that the insulin secretion was similar between AS and CTL islets (**Figure 3C**), thus excluding any major effect of ARHGAP21 knockdown on plasma cell membrane potential. These results suggest that the increased insulin secretion, in response to glucose in AS mice islets, is due to alterations in the secretory machinery and not to insulin expression or action.

Next, we explored the participation of different steps/agents of the secretory process in both AS and CTL islets. We analyzed the mRNA expression of calcium channels (CaV1.2 and SERCA) and glucose-induced intracellular Ca^2+^ alterations. Despite an increased SERCA gene expression in islets from AS mice (**Figure 4A**), the glucose/KCl-induced cell calcium dynamics was similar between islets from both groups (**Figures 4B, C**). However, the SYTVII gene expression, that encode an important Ca^2+^ sensor protein (16–18), was significantly increased in islets from AS mice (**Figure 5B**). SNAP25 gene that encode an important element for the insulin granule extrusion (19), was also significantly increased in AS islets (**Figure 5B**). Taken together these results, we believe the ARHGAP21 knockdown alters the GSIS turning the β-cell secretory machinery more sensitive to glucose, but not, by influencing the plasma cell membrane potential.

An increase in the transcript level of CX36 in islet from AS-treated mice, compared to CTL, was also observed (**Figure 5A**). CX36 forms channels (gap junctions) that allow the exchange of ions and second messengers between adjacent cells. These events are important for the synchrony of pancreatic β-cells, crucial for a proper secretory response (20, 21). Of note, the relationship between ARHGAP21 and cell junctions was already demonstrated in epithelial cells where this protein is important in the process of recruitment of α-catenin and the formation of adherent junctions (22). In addition, ARHGAP21 acts together with α-Tubulin in the formation of cell-to-cell adhesion in the epithelial-mesenchymal transition (4). Here, for the first time, we describe a possible role of ARHGAP21 in regulating the organization of gap junctions in pancreatic islets. CX36 is also involved with pancreatic islets maturation (21), however, except INS1 and INS2 genes expression, no differences in genes-related to islets maturation (NKX6, PDX1, MAFA) were observed between AS and CTL islets (**Figure 5A**). Despite the increase in the expression of INS1 and INS2 (**Figure 5A**) the total insulin content in islets from AS and CTL mice was similar (**Figure 3D**).

Reduction in PGC1α gene expression with reduction in insulin secretion and development of type 2 diabetes was already noticed (23, 24). The PGC1α seems to be important to the potentiating effect of fatty acids in the GSIS process (23). Here, we observed a higher expression of PGC1α in islets from AS, compared with CTL (**Figure 5D**), and this may explain the higher insulin secretion in the AS islets, at stimulatory glucose concentrations.

In our previous studies, we observed a possible relationship between ARHGAP21 and PGC1α pathway genes. ARHGAP21 heterozygotes mice presented an increased expression of UCP1 in BAT (Brown Adipose Tissue) (8), and increased of SREBP-1C and ACC gene expression in liver. In addition, these mice presented a reduction in CPT1a gene expression in liver (9). Therefore, based on this, we are tempted to speculate that ARHGAP21 also may affect lipids metabolism in pancreatic β-cells and consequently reduces insulin secretion.

In conclusion, ARHGAP21 seems to be an important repressor of glucose-stimulated insulin secretion in islet from adult mice through modulation of gene expression such as SYTVII, SNAP25, CX36, and PGC1α. These effects are in line with the reduction (Ob/Ob mice) or augment (trained-mice) in ARHGAP21 expression, concomitantly with increase and decrease in GSIS in these models (**Graphical Abstract**). These results corroborate previous studies showing the negatively effect of ARHGAP21 on GSIS (Ferreira et al, 2015), indicating that this protein may be important on glucose homeostasis and a possible target for new therapies for obesity-induce β-cell alterations and T2D onset.

## Data Availability Statement

The raw data supporting the conclusions of this article will be made available by the authors, without undue reservation.

## Ethics Statement

The animal study was reviewed and approved by CEUA/IB/UNICAMP – 2507-1.

## Author Contributions

SF, FO, LR, HB, and GS contributed to the research design. SF, JC-J, MK, NL, and GS conducted the experiments and acquired the data. AB provided the materials and reagents. SF, FO, LR, AB, and GS contributed to the data analysis and interpretation. SF wrote and JC-J, AB, and GS revised the manuscript. All authors contributed to the article and approved the submitted version.

## Funding

This study was supported by the São Paulo Research Foundation – FAPESP (Grant number 2011/12050-7 and 2011/09012-6).

## Conflict of Interest

The authors declare that the research was conducted in the absence of any commercial or financial relationships that could be construed as a potential conflict of interest.
