# Supplementary material for: ARHGAP21 Acts as an Inhibitor of the Glucose-Stimulated Insulin Secretion Process
Source: Front Endocrinol (Lausanne). 2020 Nov 26;11:599165. doi: 10.3389/fendo.2020.599165 (PMC7726208; doi:10.3389/fendo.2020.599165)
Supplement: Supplementary file 1 [file Table_1.pdf]

Table 01. Antibodies used for WB experiments.

Antibody	Company	Catalog #	Type	Class	Source	Reactivity
ARHGAP21 (H-300)	Santa Cruz	SC98336	Primary	Polyclonal	Rabbit	Mouse, Rat, and Human
GAPDH (FL-335)	Santa Cruz	SC25778	Primary	Polyclonal	Rabbit	Mouse, Rat, and Human
Goat anti-Rabbit IgG (H+L), HRP	Thermo Fisher/Invitrogen	31460	Secondary	Polyclonal	Goat	Rabbit
